# Prevalence and impact of metabolic syndrome on in-hospital outcomes in patients with acute myocardial infarction: A perspective from a developing country

**DOI:** 10.1097/MD.0000000000035924

**Published:** 2023-11-10

**Authors:** Nghia Thuong Nguyen, Tai Nhat Nguyen, Kha Minh Nguyen, Hai Phuong Nguyen Tran, Khoa Le Anh Huynh, Sy Van Hoang

**Affiliations:** a Department of Interventional Cardiology, Cho Ray Hospital, Ho Chi Minh City, Vietnam; b Department of Internal Medicine, Faculty of Medicine, University of Medicine and Pharmacy at Ho Chi Minh City, Ho Chi Minh City, Vietnam; c Department of Cardiology, Cho Ray Hospital, Ho Chi Minh City, Vietnam; d Department of Biostatistics, Virginia Commonwealth University School of Medicine, VA, USA.

**Keywords:** acute myocardial infarction, cardiovascular mortality, metabolic syndrome

## Abstract

Acute myocardial infarction (AMI) often suffers from a high prevalence of metabolic syndrome (MetS). However, few studies in developing countries have focused on the effect of MetS on in-hospital outcomes in patients with AMI. We analyzed 199 patients with AMI who underwent primary percutaneous coronary intervention. This study aimed to determine the impact of MetS and factors related to in-hospital outcomes in patients with AMI. The study included 199 patients who met the criteria, with a mean age of 64.5 ± 11.3 years. Out Of the total number of patients, 136 (68.3%) were found to have MetS. Patients with MetS were more likely to be female, have a higher body mass index, larger waist circumference, and a higher prevalence of hypertension and diabetes than those without MetS. The rates of major complications, such as cardiogenic shock, heart failure, mechanical complications, and arrhythmias, were not significantly different between the 2 groups. MetS was not associated with in-hospital mortality with OR, 4.92 (95% CI 0.62–39.31, *P* = .13). In this study, increased waist circumference was associated with an increased all-cause mortality rate. However, the MetS group had a significantly higher rate of cardiovascular mortality than the group without MetS (*P* = .03). Among patients with AMI, the prevalence of metabolic syndrome was high. Patients with MetS did not exhibit an increased all-cause in-hospital mortality rate. Increased waist circumference is associated with increased all-cause mortality.

## 1. Introduction

Metabolic syndrome (MetS) is a group of risk factors that include high blood glucose levels and dyslipidemia that can cause atherosclerosis, central obesity, high blood pressure, and pre-thrombotic and pro-inflammatory conditions.^[1–3]^ MetS is associated with an increased risk of coronary artery disease, cardiovascular disorders, and diabetes mellitus type 2.^[4–7]^ It is estimated that MetS affects approximately 25% of the global population and is becoming a significant health concern in the world.^[8]^ Recent research has shown that the prevalence of MetS in patients with acute myocardial infarction (AMI) is exceptionally high in developed and Eastern countries. Moreover, the occurrence of MetS is more prevalent in populations with an increased risk of cardiovascular diseases such as acute coronary syndrome, stroke, and peripheral artery disease.^[9]^ The rising prevalence of MetS in Asia-Pacific nations is causing worry that it could pose a significant public health issue that demands immediate action.^[10,11]^ Until around 20 years ago, the prevalence of MetS in Asian countries was low compared with that in Western countries.^[12,13]^

A comprehensive meta-analysis of 87 studies involving 951,083 patients demonstrated that MetS is associated with a 2-fold increase in cardiovascular mortality, AMI, and stroke, and a 1.5-fold increase in all-cause mortality risk.^[14]^ Most of these studies were carried out in developed countries among patients with ACS who were managed differently, and this likely affected their prognosis and the likelihood of new adverse outcomes. However, the link between MetS and the probability of cardiovascular disease and mortality risk varies by race/ethnicity, age, geographical area, and healthcare accessibility.^[9,14]^ Limited data exist on the association between MetS and the prognosis of patients with ACS in developing countries, especially those who have received primary percutaneous coronary intervention.

Therefore, we sought to evaluate the prevalence and impact of MetS on in-hospital mortality among patients hospitalized with ACS who underwent percutaneous coronary intervention in a tertiary hospital in Vietnam.

## 2. Methods

### 2.1. Study design and population

This prospective cohort study was conducted at the Department of Interventional Cardiology, Cho Ray Hospital, between November 2022 and May 2023. The inclusion criteria were as follows: age ≥18 years, diagnosis of AMI type I according to the Fourth Universal Definition of Myocardial Infarction based on coronary angiography,^[15]^ and willingness to participate in the study. The exclusion criteria were secondary hyperlipidemia, secondary hypertension, and statin use within 4 weeks before hospital admission. The patients in the study were diagnosed and treated based on the national guidelines of the Vietnam Ministry of Health for AMI. The study was approved by the Ethics Committee for Biomedical Research at the University of Medicine and Pharmacy, Ho Chi Minh City ([ID:22592-DHYD] on 31st October 31, 2022) and written consent was obtained from all patients.

### 2.2. Study variables

Metabolic syndrome is diagnosed based on consensus on the definition of MetS for the Asian population published in 2009, with the endorsement of IDF, AHA, ACC, WHF, IAS, and IASO.^[16]^ The syndrome is considered to be present when at least 3 out of 5 criteria are met: increased waist circumference (≥80 cm in females or ≥90 cm in males), elevated blood pressure (SBP ≥130 mm Hg and/or DBP ≥85 mm Hg, or currently undergoing treatment for hypertension in patients with a history of high blood pressure), elevated fasting blood glucose (fasting blood glucose ≥100 mg/dL or currently on diabetes treatment), elevated triglycerides (≥150 mg/dL or currently on lipid-lowering treatment for elevated triglycerides), Reduced high-density lipoprotein (HDL) cholesterol (<40 mg/dL in males or <50 mg/dL in females, or currently on lipid-lowering treatment for reduced HDL cholesterol).

Waist circumference was calculated as the average of 2 measurements at the midpoint between the lower rib margin and the top of the iliac crest in the horizontal plane. The patient stood upright and measurements were taken at the end of normal exhalation. Blood pressure measurements were obtained while the patient was seated and the average of 3 readings was recorded. Blood samples were collected from a fasting vein in the morning after 8 to 12 hours of fasting overnight. This was done to measure fasting plasma glucose, HDL-c, low-density lipoprotein-c, triglycerides, and total cholesterol levels.^[17]^

### 2.3. Clinical outcome

The investigator recorded all-cause death during the hospital stay period. Cardiovascular death was assessed based on international guidelines. Cardiovascular death included deaths from sudden cardiac arrest, heart failure, stroke, cardiovascular procedures, and other related causes. All complications of AMI were adjudicated centrally by 2 independent cardiologists, and any disagreement was resolved by consensus.

### 2.4. Statistical analysis

Data entry and processing were conducted using Stata 14.2 software on a Windows operating system (StataCorp. 2015. Stata Statistical Software: Release 14. College Station, TX, StataCorp LP). The normality check of the numerical variables was performed using the Shapiro–Wilk test. Continuous variables with a normal distribution were described as mean ± standard deviation. If the distributions were not normal, they were described using the median (25th–75th quartiles). Categorical and ordinal variables are defined as frequencies and percentages. Differences in means between groups were compared using the *t* test for normally distributed variables and the Mann–Whitney *U* test for non-normally distributed variables. Differences in the frequency distributions of categorical variables were assessed using the chi-square test (χ²) or Fisher exact test.

A logistic regression model was used to assess the relationship between MetS and in-hospital mortality. The initial model included age, sex (female), serum creatinine clearance, ST-elevation myocardial infarction (STEMI), smoking, vital signs at admission (including heart rate, systolic blood pressure, and diastolic blood pressure), previous MI, and MetS itself as predictors of cardiac and overall mortality. These independent variables were selected owing to their significant prognostic value in acute coronary syndrome.^[18]^ The Chi-squared test was used to assess statistically significant differences. Adjusted odds ratios (OR) and 95% confidence intervals were reported. Relative risk with a 95% confidence interval was used to evaluate the relationship between factors in the MetS and outcomes. Statistical significance was set at *P* value < .05 was considered statistically significant.

## 3. Results

### 3.1. Clinical characteristics

**Figure 1.**
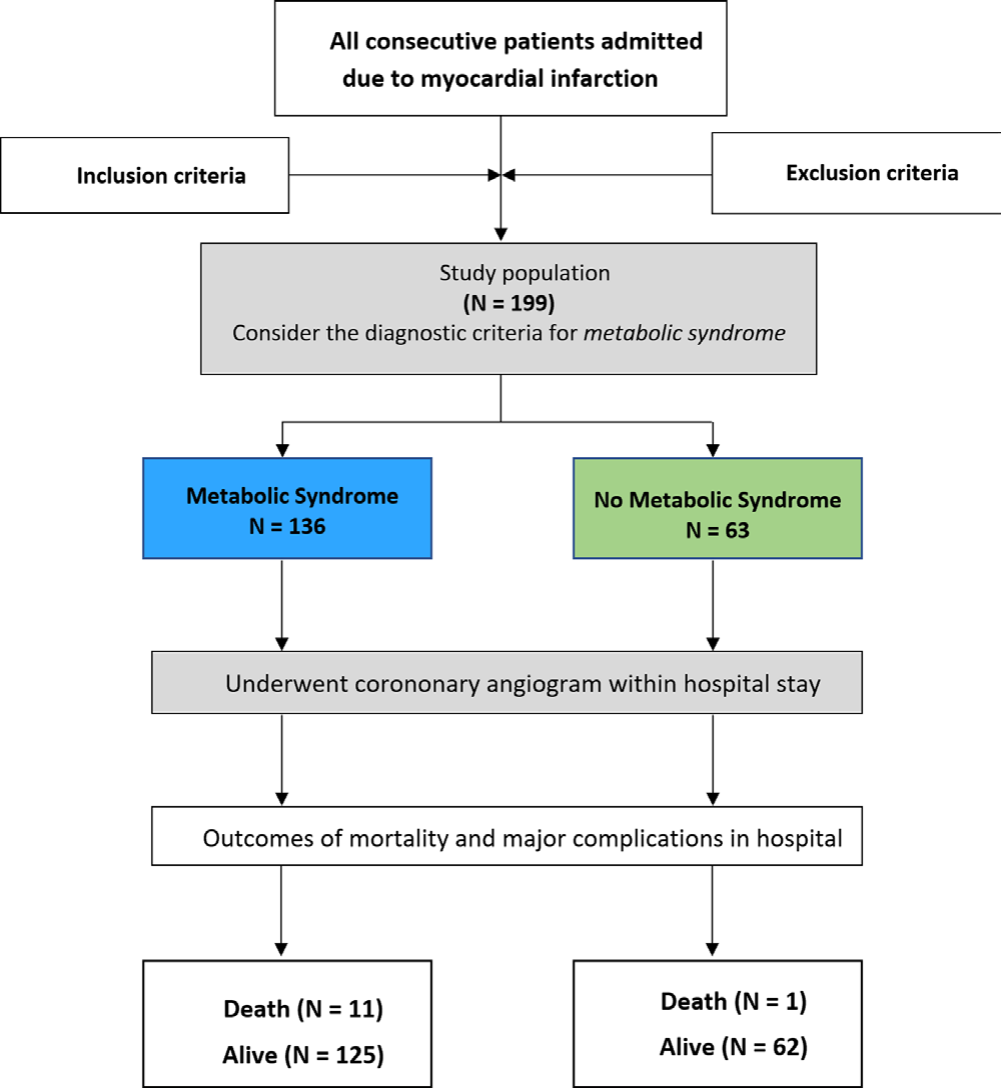
The flow chart of study population.

**Figure 2.**
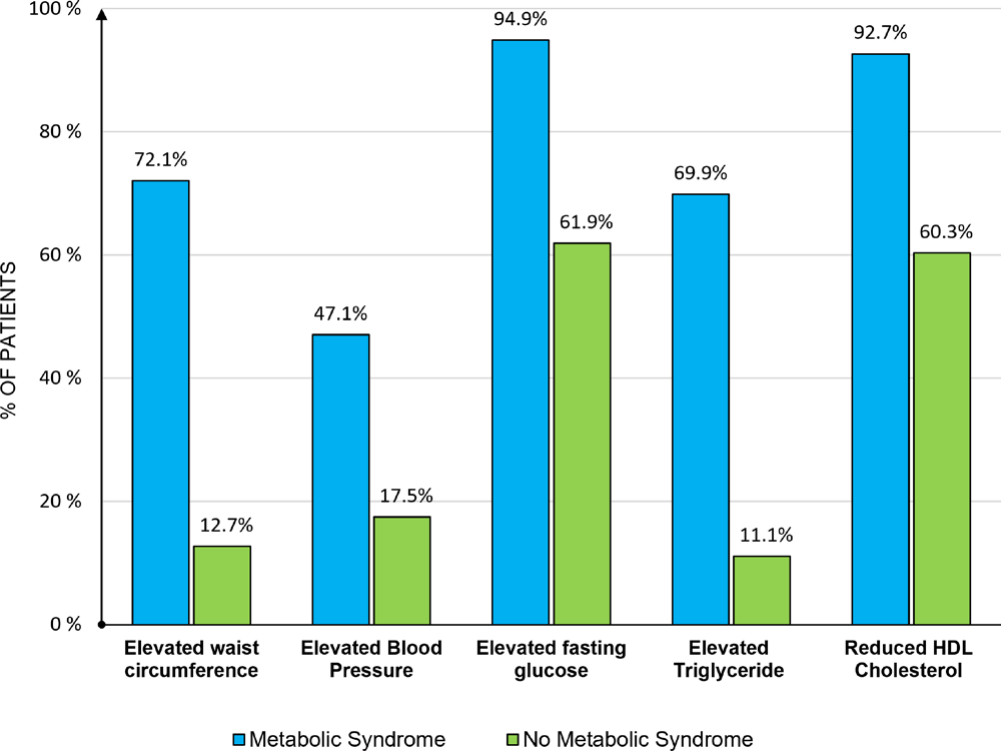
Frequency of components in the metabolic syndrome diagnostic criteria in the study.

We enrolled 199 patients with AMI who met the inclusion criteria from November 2022 to May 2023 (Fig. 1). Within the study population, 136 patients (68.3%) met the criteria for diagnosing MetS. The frequency distributions of the components of the MetS diagnostic criteria are shown in Figure 2.

The baseline characteristics of the study population in the 2 groups with and without MetS are shown in Table 1. The mean age of the study population was 64.5 ± 11.3 years, ranging from 41 to 91 years, with no significant difference between the 2 groups. Patients with MetS had a higher proportion of females than the non-MetS group with 31.6% and 15.9%, respectively (*P* = .02). Additionally, the MetS group had a higher body mass index (BMI), larger waist circumference, and higher proportions of hypertension and diabetes than the non-MetS group (*P* values < .05. There was no significant difference between the 2 groups regarding the Killip class at admission and Killip class ≥ II.

**Table 1:** Baseline clinical characteristics of the study population.

Characteristic	All (N = 199)	MetS (+) (N = 136)	MetS (−) (N = 63)	*P* value
Age, yr	64.5 ± 11.3	64.5 ± 11.7	64.6 ± 10.4	.52
Male sex, n (%)	146 (73.4%)	93 (68.4%)	53 (84.1%)	**.02**
Female sex, n (%)	53 (26.6%)	43 (31.6%)	10 (15.9%)	
BMI, (kg/m^2^)	23.2 ± 3.1	23.9 ± 2.8	21.7 ± 3.1	**<.001**
Waist circumference, cm	89.1 ± 8.6	91.5 ± 8.1	83.8 ± 7.0	**<.001**
Cardiovascular history				
CAD, n (%)	16 (8.0%)	11 (8.1%)	5 (7.9%)	.97
Current smoker, n (%)	85 (42.7%)	52 (38.2%)	33 (52.4%)	.06
Hypertension, n (%)	63 (31.7%)	55 (40.4%)	8 (12.7%)	**<.001**
Diabetes mellitus, n (%)	42 (21.1%)	38 (27.9%)	4 (6.4%)	**<.001**
Heart failure, n (%)	1 (0.5%)	1 (0.7%)	0 (0%)	.50
Previous PCI, n (%)	4 (2.0%)	3 (2.2%)	1 (1.6%)	1.00
Previous CABG, n (%)	0 (0%)	0 (0%)	0 (0%)	-
Dyslipidemia, n (%)	11 (5.5%)	10 (7.4%)	1 (1.6%)	.10
Previous MI, n (%)	14 (7.0%)	10 (7.4%)	4 (6.4%)	.80
Clinical data				
Killip classification				
I, n (%)	146 (73.4%)	96 (70.6%)	50 (79.4%)	.48
II, n (%)	24 (12.1%)	18 (13.2%)	6 (9.5%)	
III, n (%)	12 (6.0%)	8 (5.9%)	4 (6.4%)	
IV, n (%)	17 (8.5%)	14 (10.3%)	3 (4.8%)	
Killip ≥ II, n (%)	53 (26.6%)	40 (29.4%)	13 (20.6%)	.19
Heart rate, bpm	79.8 ± 18.2	81.9 ± 17.3	75.3 ± 19.3	**.01**
SBP, mm Hg	120.7 ± 25.0	123.5 ± 26.9	114.5 ± 19.1	**.02**
DBP, mm Hg	73.8 ± 14.4	75.4 ± 15.1	70.3 ± 12.0	**.02**
PCI, n (%)	197 (99.0%)	135 (99.3%)	62 (98.4%)	.53
Myocardial infarction				
STEMI, n (%)	144 (72.4%)	94 (69.1%)	50 (79.4%)	.13
NSTEMI, n (%)	55 (27.6%)	42 (30.9%)	13 (20.6%)	
LVEF classification				
EF ≤ 40%, n (%)	99 (49.8%)	69 (50.7%)	30 (47.6%)	.68
EF 41–49%, n (%)	39 (19.6%)	25 (18.4%)	14 (22.2%)	.53
EF ≥ 50%, n (%)	61 (30.7%)	42 (30.9%)	19 (30.2%)	.92
LVEF, %	42.2 ± 11.5	42.1 ± 11.4	42.3 ± 11.7	.91

Values were presented as mean ± Standard deviation or frequency (percentage). Bold characters refer to statistical differences.

BMI = body mass index, CABG: = coronary artery bypass graft surgery, CAD = coronary artery disease, DBP = diastolic blood pressure, LVEF = left ventricular ejection fraction, MetS = metabolic syndrome, MI = myocardial infarction, NSTEMI = non-ST-segment elevation myocardial infarction, PCI = percutaneous coronary intervention, SBP = systolic blood pressure, SD = standard deviation, STEMI = ST-segment elevation myocardial infarction.

Clinical laboratory tests in the MetS group revealed significant differences in the following parameters compared to those without MetS (Table 2): fasting blood glucose, triglyceride, and total cholesterol levels were higher in the MetS group. HDL-C levels were significantly lower in the MetS group. No significant difference was observed in low-density lipoprotein-C levels between the 2 groups.

**Table 2:** In-hospital clinical features in the study population.

Characteristic	All (N = 199)	MetS (+) (N = 136)	MetS (−) (N = 63)	*P* value
eGFR, mL/min/1.73 m^2^	74.4 ± 22.0	74.6 ± 21.6	74.2 ± 23.2	.45
eGFR < 60 mL/min, n (%)	54 (27.1)	35 (25.7)	19 (30.2)	.51
Fasting glucose, mg/dL	162.5 ± 79.9	173.7 ± 79.6	138.5 ± 75.6	**.004**
Triglycerid, mg/dL	190.0 ± 125.0	221.1 ± 134.4	123.0 ± 62.4	**<.001**
HDL-C, mg/dL	34.5 ± 9.0	32.8 ± 8.1	38.1 ± 9.8	**<.001**
LDL-C, mg/dL	121.6 ± 45.4	121.7 ± 44.9	121.6 ± 47.0	.50
Total Cholesterol, mg/dL	194.1 ± 50.3	198.7 ± 50.4	184.3 ± 49.0	**.03**
Troponin I hs, pg/mL	21610.8 ± 21518.3	21375.1 ± 22204.9	22119.5 ± 20118.4	.59

Values were presented as mean ± Standard deviation or frequency (percentage). Bold characters refer to statistical differences.

eGFR = estimated glomerular filtration rate, HDL = high-density lipoprotein, hs = high sensitivity, LDL = low-density lipoprotein, MetS = metabolic syndrome.

**Figure 3.**
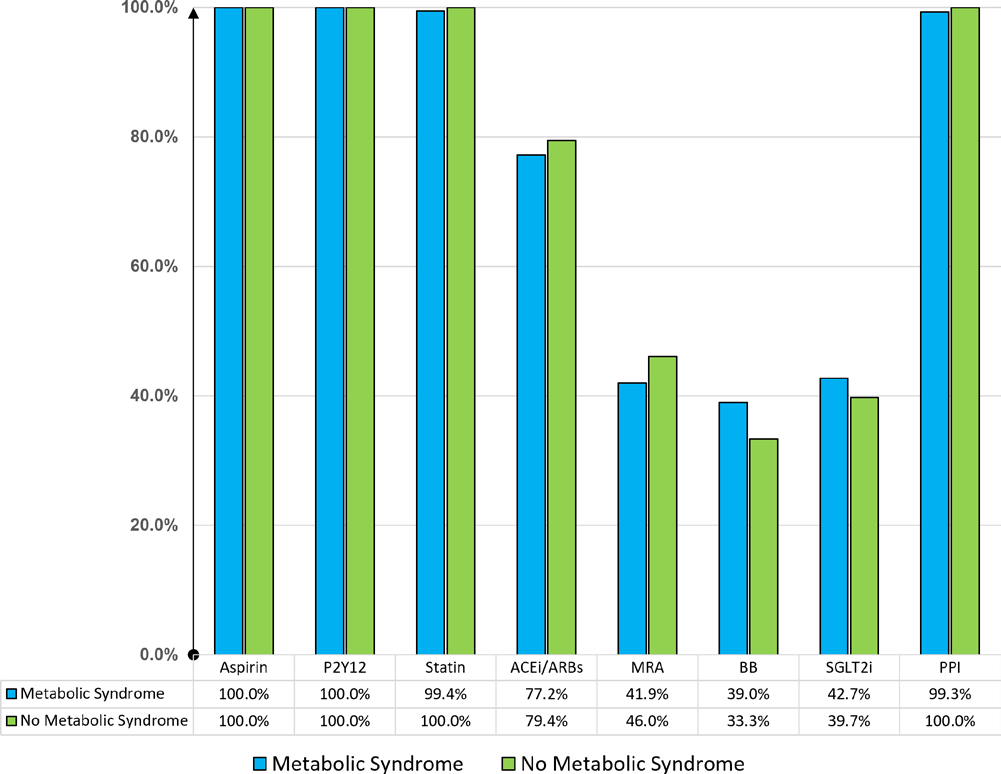
Outcome of mortality and major in-hospital complications in the study.

Evaluation of left ventricular systolic function in our study was performed using the left ventricular ejection fraction calculated using Simpson method in a 4-chamber view. The average left ventricular ejection fraction was 42.10 ± 11.4 % in the MetS group and 42.3 ± 11.7 % in the non-MetS group, with no significant difference observed (*P* = .91). Left ventricular systolic function reduction, defined as EF ≤ 40%, was not statistically different between the 2 groups (*P* = .68). Figure 3 shows the percentage of medications used by the study population, and there were no significant differences between the 2 groups.

### 3.2. In-hospital outcomes and metabolic syndrome

All-cause death in the group with MetS was 7.4%, which was higher compared to the group without MetS (Table 3). However, this difference was not statistically significant (*P* = .18). When considering the criteria for cardiac mortality, the MetS group showed a considerable difference compared to the non-MetS group (*P* = .03). There were no significant differences in other major in-hospital complications.

**Table 3:** The outcome of mortality and major in-hospital complications.

Outcomes	All (N = 199)	MetS (+) (N = 136)	MetS (−) (N = 63)	*P* value
All-cause death, n (%)	11 (5.5%)	10 (7.4%)	1 (1.6%)	.18
Cardiovascular death, n (%)	10 (5.0%)	10 (7.4%)	0 (0%)	**.03**
Major complications in hospital				
Cardiogenic shock, n (%)	24 (12.1%)	19 (14.0%)	5 (7.9%)	.22
Heart failure, n (%)	111 (55.8%)	75 (55.2%)	36 (57.1%)	.79
Mechanical complications, n (%)	4 (2.0%)	3 (2.2%)	1 (1.6%)	.77
Ventricular tachycardia/Ventricular fibrillation, n (%)	4 (2.0%)	4 (2.9%)	0 (0%)	NA
Bradycardia required TPM, n (%)	15 (7.5%)	10 (7.4%)	5 (7.9%)	1.00
Stroke, n (%)	0 (0%)	0 (0%)	0 (0%)	NA
Reinfarction, n (%)	1 (0.5%)	1 (0.7%)	0 (0%)	1.00
Repeated PCI, n (%)	1 (0.5%)	1 (0.7%)	0 (0%)	1.00

Values were presented as frequency (percentage). Bold characters refer to statistical differences.

MetS = metabolic syndrome, PCI = percutaneous coronary intervention, TPM = temporary pacemaker.

### 3.3. Predictors of in-hospital all-cause mortality

We conducted a univariate logistic regression analysis to assess the relationship between MetS and the prediction of in-hospital mortality, adjusted for age, sex, eGFR, STEMI, smoking status, heart rate, systolic blood pressure, diastolic blood pressure at admission, Killip class ≥ II, and prior myocardial infarction (Table 4). When analyzing the relationship between MetS components and all-cause mortality, it was found that increased waist circumference was strongly associated with all-cause mortality (Table 5).

**Table 4:** The univariable logistic regression model predicts the outcome of all-cause mortality.

Components	All-cause death		
	OR	Confidence interval 95%	*P* value
Metabolic syndrome	4.92	0.62–39.31	.13
Age	1.00	0.95–1.06	.90
Female sex	0.411	0.12–1.41	.16
eGFR < 60 mL/min	5.25	1.47–18.73	**.01**
STEMI	1.00	-	-
Smoker	0.28	0.06–1.33	.11
KILLIP ≥ II	14.73	3.06–70.71	**.001**
Pulse	1.00	0.97–1.04	.70
SBP	0.97	0.94–0.99	**.003**
DBP	0.97	0.94–1.01	.12
Previous MI	1.00	-	-

Bold characters refer to statistical differences.

DBP *=* diastolic blood pressure, eGFR *=* estimated glomerular filtration rate, MI = myocardial infarction, SBP *=* systolic blood pressure, STEMI *=* ST-segment elevation myocardial infarction.

**Table 5:** The association between the components of metabolic syndrome and all-cause mortality.

Components	Death group (N = 11)	Non-death group (N = 188)	RR (Confidence interval 95%)	*P* value
Elevated fasting glucose	11 (100%)	157 (83.5%)	+ Infinity	.14
Elevated blood pressure	1 (9.1%)	74 (39.4%)	0.15 (0.02–1.23)	.08
Elevated waist circumference	11 (100%)	95 (50.5%)	+ Infinity	**.001**
Elevated triglyceride	3 (27.3%)	99 (52.7%)	0.36 (0.10–1.31)	.10
Reduced HDL-C	9 (81.8%)	155 (82.5%)	0.96 (0.22–4.25)	.96

Bold characters refer to statistical differences.

HDL-C = high-density lipoprotein cholesterol, RR = risk ratio.

## 4. Discussion

In our study, the prevalence of MetS in patients with AMI was 68.3%. In patients with established CVD, the prevalence of MetS ranged from 29% to 66% in previous reports.^[9,19–22]^ In Taiwan, Li-Hong Zhao et al (2022) reported a prevalence of MetS of 50.9%.^[13]^ Similarly, Lee et al reported a 59.40% prevalence of ST-elevation AMI in South Korea.^[23]^ These findings suggest that MetS is a common occurrence in the population of coronary artery disease patients, particularly among those with AMI.

The group of patients with MetS had a higher rate of all-cause mortality than those without MetS; however, this difference was not statistically significant (*P* = .18). Interestingly, the group with MetS had a significantly higher rate of cardiac mortality than the non-MetS group (*P* = .03). According to Lovic et al’s study in Serbia, there was no statistically significant difference in overall mortality rates between the MetS group (5.99%) and the non-MetS group (6.20%) (*P* = .92).^[24]^ This study also had a relatively small sample size of only 507 patients. Several other studies showed that patients with AMI and MetS had a higher in-hospital mortality rate than those without MetS.^[23,25,26]^ In a study of 1990 Korean patients, Lee et al found that those with AMI and MetS had a statistically significant increase in in-hospital mortality (*P* = .047).^[23]^ In another study conducted in India with a sample size similar to our study (197 vs 199 patients), the acute MI patient presence of MetS was associated with about 4 times more chances of complications, including death, than those without MetS. However, they also did not analyze in depth any meaningful differences that may be related to the low number of in-hospital events. In our study, all-cause mortality in the group with metabolic syndrome was about 4 times higher than in the group without MetS, and the difference was insignificant (7.4% vs 1.6%; *P* = .18).^[25]^ A study performed in France revealed that MetS is associated with an increased mortality rate. However, after adjusting for critical factors in determining mortality among AMI patients, MetS ceased to be an independent predictor of mortality (*P* = .41). The authors explained this outcome by the possibility of a small sample size leading to a lower statistical power.^[26]^ A pooled analysis involving 45 studies and 145.897 patients demonstrated that MetS was associated with a higher cardiovascular mortality rate than the non-MetS group, with a relative risk of 1.36 (95% CI: 1.15–1.61) and *P* < .01.^[27]^ Therefore, the study sample size may be a crucial factor related to the statistically significant difference in in-hospital outcomes for patients with or without MetS.

In the univariable logistic regression analysis aimed at identifying factors related to all-cause in-hospital mortality, MetS exhibited an OR of 4.92 (95% CI: 0.62–39.31) with a *P* value of .13, indicating no significant association with in-hospital all-cause mortality. However, we observed that among patients with AMI who exhibited heart failure symptoms with a Killip class of II or higher, there was a significant increase in in-hospital all-cause mortality, with an OR of 14.73 (95% CI: 3.06–70.71) and a *P* value of .001. Heart failure is a common complication of AMI, with frequencies varying from 14% to 36%, depending on the study. Heart failure strongly predicts mortality among AMI patients and significantly influences treatment strategies.^[28]^ Despite variations in the definitions of heart failure used across studies, many include patients with Killip class II (with lung rales or a third heart sound) and Killip class III (acute pulmonary edema), both indicative of heart failure. The Killip classification describes the relative severity of heart failure and is a strong predictor of mortality.^[29]^ Additionally, renal function with eGFR <60 mL/minute/1.73 m² (CKD-EPI) was found to increase the all-cause in-hospital mortality rate, with an OR of 5.25 (95% CI: 1.47–18.73) and *P* value of .01. Impaired renal function in patients with ST-elevation myocardial infarction is also well established as a predictor of both in-hospital and long-term mortality.^[30,31]^ According to a study conducted in Egypt, patients with STEMI and an eGFR of <60 mL/minute are 4 times more likely to experience mortality than those with an eGFR >60 mL/minute (*P* < .001).^[32]^ This finding could be explained by decreased renal function often associated with advanced age, hypertension, and diabetes. Furthermore, renal pathologies can increase arterial stiffness and calcification, affecting multiple organs and limiting medications such as ACE inhibitors or ARBs.^[33]^

When analyzing each component of the diagnostic criteria for MetS regarding in-hospital mortality outcomes, we observed that increased waist circumference (≥90 cm in males and ≥80 cm in females) was associated with in-hospital all-cause mortality (*P* = .001). Other criteria did not show significant associations, including elevated triglycerides (*P* = .10), reduced HDL-C (*P* = .96), elevated fasting blood glucose (*P* = .14), and elevated blood pressure (*P* = .08). The association of increased waist circumference or central obesity, which partly reflects visceral fat accumulation, with MetS, dyslipidemia, inflammatory status, insulin resistance, and diabetes, is related to an increased risk of cardiovascular diseases. Compared to the BMI index, central obesity indicators such as waist circumference or waist-to-hip ratio correlate more strongly with cardiovascular events and cardiac mortality.^[34]^ Bakhoum et al found no significant impact of waist circumference on in-hospital mortality in patients with myocardial infarction. The waist circumference index was calculated as increased or not (OR = 0.374, 95% CI: 0.01–5.0, *P* = .374).^[35]^ Zeller et al (2008) found that BMI and waist circumference were not independent predictors of mortality following myocardial infarction.^[36]^ Furthermore, Koning et al conducted a meta-analysis of 15 prospective studies, which showed that an increased waist circumference or waist-to-hip ratio increased the risk of cardiovascular events in both men and women, even after adjusting for age and population characteristics.^[37]^

Regarding major cardiac complications during hospitalization, the rates of complications in the group with MetS and the group without MetS were as follows: cardiogenic shock (14.0% and 7.9% with *P* = .22), heart failure (55.2% and 57.1%, *P* = .79), mechanical complications (2.2% and 1.6% with *P* = .77), and ventricular arrhythmias (2.9% and 0% with *P* = .31); all showed no statistically significant differences. In summary, major in-hospital cardiac complications in our study did not show significant differences between groups with and without MetS. Lovic et al conducted a survey in Serbia in 2018 to investigate the major in-hospital complications in patients with myocardial infarction. The study found that there were no significant differences between the 2 groups in terms of complications such as heart failure based on Killip classification II or higher, ventricular arrhythmias (ventricular tachycardia, ventricular fibrillation), conduction disorders, or stroke.^[24]^ Our findings align with these results.

Our study has several limitations. First, the convenient sampling technique and limited study duration resulted in a relatively small study population. This limitation may influence our study negative association between metabolic syndrome and in-hospital outcomes of patients with AMI. Additionally, as this was a single-center study conducted at the cardiology center of Cho Ray Hospital, the study population might only partially represent the research population in Vietnam. Therefore, further large-scale, multicenter studies are needed to comprehensively characterize MetS in AMI and to determine the impact of MetS on the severity of coronary anatomy as well as its association with mortality and major in-hospital complications.

## 5. Conclusion

Our study revealed that patients with AMI had a higher prevalence of metabolic syndrome. Patients with metabolic syndrome had a higher prevalence of hypertension, diabetes, and obesity, particularly among females with larger waist circumferences. The overall mortality rate for all causes did not differ significantly between the 2 groups. Increased waist circumference is associated with increased all-cause mortality.

## Acknowledgments

This study was conducted at the Interventional Cardiology Department of the Cho Ray Hospital. We would like to thank the medical staff who helped with data collection.

## Author contributions

**Conceptualization:** Nghia Thuong Nguyen, Hai Phuong Nguyen Tran, Sy Van Hoang.

**Data curation:** Tai Nhat Nguyen, Kha Minh Nguyen, Hai Phuong Nguyen Tran, Sy Van Hoang.

**Formal analysis:** Nghia Thuong Nguyen, Tai Nhat Nguyen, Kha Minh Nguyen, Hai Phuong Nguyen Tran, Khoa Anh Le Huynh.

**Investigation:** Nghia Thuong Nguyen, Hai Phuong Nguyen Tran, Sy Van Hoang.

**Methodology:** Kha Minh Nguyen, Khoa Anh Le Huynh.

**Software:** Kha Minh Nguyen, Khoa Anh Le Huynh.

**Supervision:** Nghia Thuong Nguyen, Sy Van Hoang.

**Writing – original draft:** Nghia Thuong Nguyen, Tai Nhat Nguyen, Kha Minh Nguyen, Sy Van Hoang.

**Writing – review & editing:** Nghia Thuong Nguyen, Tai Nhat Nguyen, Kha Minh Nguyen, Sy Van Hoang.
